# Clinical Management of a Peri-Implant Giant Cell Granuloma

**DOI:** 10.1155/2015/976756

**Published:** 2015-12-14

**Authors:** A. Pacifici, D. Carbone, R. Marini, G. L. Sfasciotti, L. Pacifici

**Affiliations:** Department of Oral and Maxillofacial Science, “Sapienza” University of Rome, Via Caserta 6, 00161 Rome, Italy

## Abstract

*Purpose*. Implant therapy plays an important role in contemporary dentistry with high rates of long-term success. However, in recent years, the incidence of peri-implantitis and implant failures has significantly increased. The peripheral giant cell granuloma (PGCG) rarely occurs in peri-implant tissues and it is clinically comparable to the lesions associated with natural teeth. Therefore, the study of possible diseases associated with dental implants plays an important role in order to be able to diagnose and treat these conditions.* Materials and Methods*. This report described a 60-year-old Caucasian male who presented a reddish-purple pedunculated mass, of about 2 cm in diameter, associated with a dental implant and the adjacent natural tooth.* Results*. An excisional biopsy was performed and the dental implant was not removed. Histological examination provided the diagnosis of PGCG. After 19-month follow-up, there were no signs of recurrence of peri-implantitis around the implant.* Conclusion*. The correct diagnosis and appropriate surgical treatment of peri-implant giant cell granuloma are very important for a proper management of the lesion in order to preserve the implant prosthetic rehabilitation and prevent recurrences.

## 1. Introduction

Peripheral giant cell granuloma (PGCG) is an exophytic lesion of the gingiva, arising from the periosteum or from the periodontal membrane in response to local irritating factors or a chronic trauma [1–3]. It clinically appears as a reddish-purple sessile or pedunculated mass with soft consistency and smooth or ulcerated surface.

PGCG mainly affects female patients (60% of cases) and, although it can occur at any age, has a peak incidence between 40 and 60 years [3, 4]. It is most commonly located in the lower arch (64% of cases), especially in the gingiva of the premolars area (43% of cases) [1, 4, 5].

The maximum capacity of expansion of PGCG is unknown because it is usually surgically removed in early proliferative stages, but lesions of maximum 5 cm diameter in patients with poor oral hygiene or xerostomia have been reported [6]. The PGCG is usually asymptomatic, although it can undergo ulceration after traumas due to interference with mastication, causing bleeding and pain. In some cases, there may be bone resorption in the area of the lesion or involvement of the supporting tissues of the adjacent teeth which appears on the radiography as a flare of the periodontal ligament [2, 3, 7].

The etiopathogenesis of PGCG is still uncertain: the lesion is thought to be a reactive localized tissue response towards several possible etiologic factors as dental plaque, calculus, tooth extractions, denture irritation, periodontitis, food impaction, orthodontic therapy, inadequate restorations, ill-fitting dentures, and hormonal disturbances, like hyperparathyroidism [8–10].

The treatment of PGCG includes surgical excision and elimination of the possible etiologic factors [11, 12].

Histologically, PGCGs show the presence of nonencapsulated highly cellular mass with abundant multinucleated giant cells, inflammatory cells, interstitial haemorrhage, hemosiderin deposits, and mature bone [4]. Some authors think these multinucleated giant cells to be formed by the fusion of osteoclasts while others report an origin from macrophages [13–17].

PGCGs associated with dental implants are very rare. The first case was described by Hanselaer et al. in 1984 [18] and only 15 cases are currently reported in the literature [19–29] (Table 1).

The aim of this paper is to report a successful clinical and surgical management of PGCG associated with a dental implant and the adjacent natural tooth.

## 2. Case Report

A 60-year-old Caucasian man referred to the Department of Oral and Maxillofacial Science, “Sapienza” University of Rome, with a chief complain of swelling in the anterior zone of the palate. His medical history was negative and he was not taking drugs at the time.

**Figure 1:**
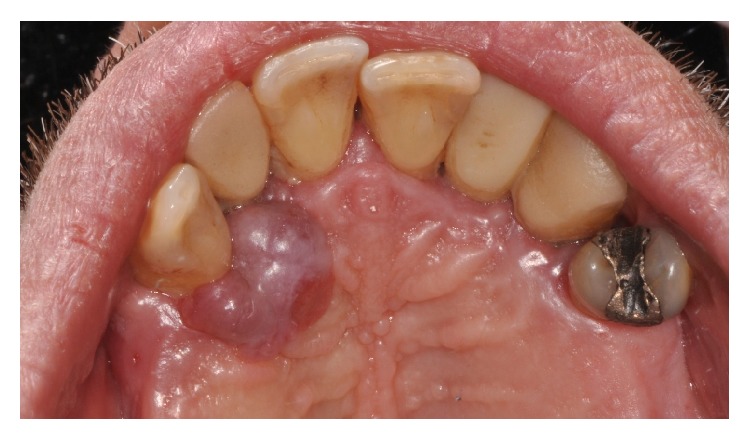
Intraoral examination: note the exophytic mass on the palatal aspect associated with the canine and dental implant.

**Figure 2:**
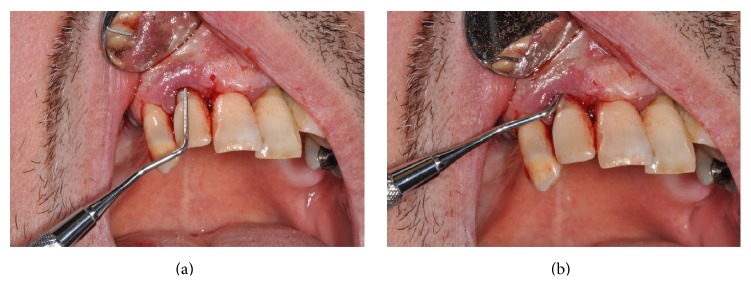
(a, b) Peri-implant probing.

Intraoral examination revealed a nonulcerated red-purple pedunculated mass, of about 2 cm in diameter, with elastic consistency and smooth erythematosus surface, arising from the gingival margin of the anterior right palatal region (Figure 1). The lesion was extended from the distal side of the upper right lateral incisor to the distal side of the upper right canine. The lateral incisor was replaced five years before with a dental implant. The oral hygiene appeared to be poor. Furthermore, a probing depth of 10 mm was recorded vestibular to the implant (Figures 2(a) and 2(b)). Contradictorily, there were no signs of implant or dental mobility and the patient referred no symptomatology.

**Figure 3:**
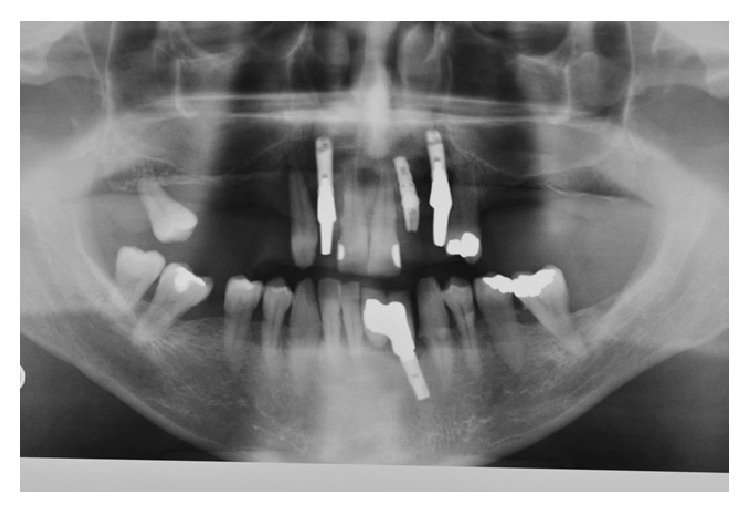
Panoramic radiograph showing a radiolucent lesion surrounding the implant and the upper right canine.

The radiographic evaluation (Figure 3) revealed a large radiolucent area that involved the implant and element 1.3.

The diagnostic hypotheses of peri-implant infection, granuloma, and trauma reaction were carried out and an excisional biopsy was programmed, with the decision of maintaining dental implant.

**Figure 4:**
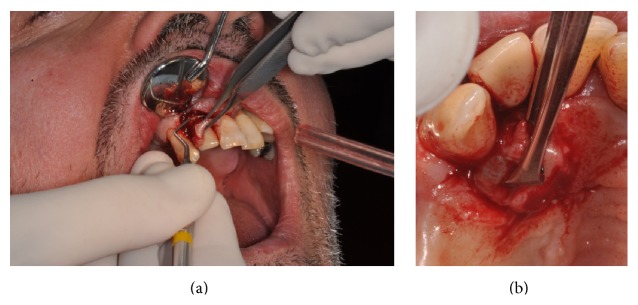
(a, b) Vestibular and palatal excisional biopsy of the lesion.

**Figure 5:**
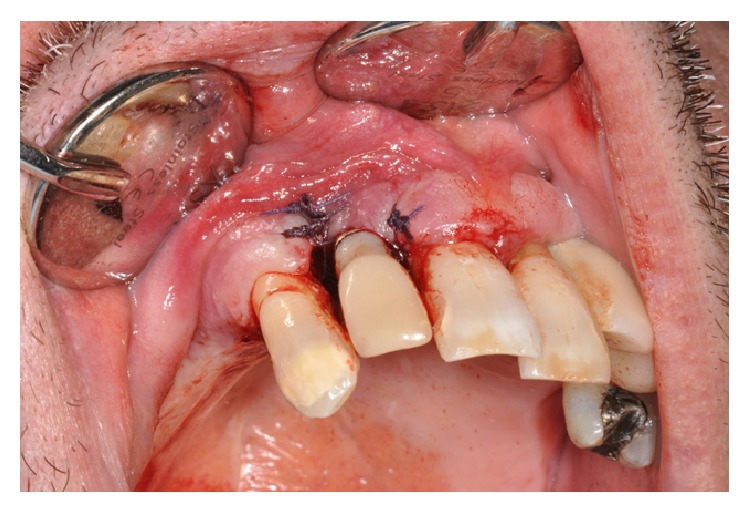
Suture of the surgical wound.

Vestibular and palatal excisional scalpel biopsy was performed in local anaesthesia (Figures 4(a) and 4(b)), with an accurate curettage of the remaining surgical site, followed by the application of an absorbable suture (Vicryl 4/0, Johnson & Johnson, Sint-Stevens-Woluwe, Belgium) (Figure 5).

**Figure 6:**
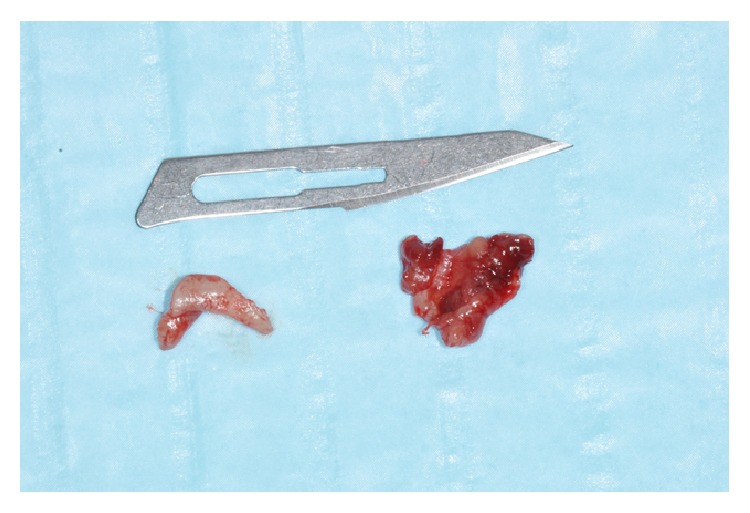
Histologic specimens.

The specimens were fixed in 10% formalin solution for histologic examination (Figure 6).

**Figure 7:**
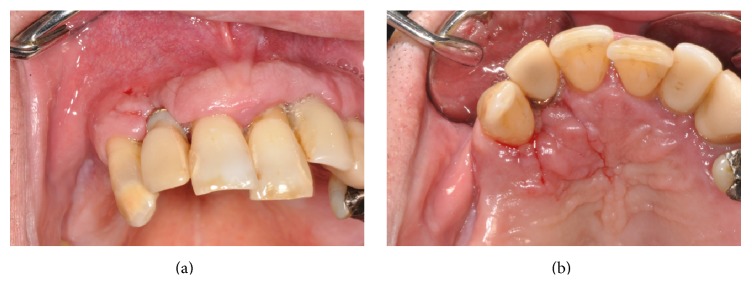
(a, b) Follow-up after 7 days.

There were no complications in the postoperative period and the clinical examination of the surgical site showed good wound healing after 7 days (Figures 7(a) and 7(b)) and complete healing without any symptoms after 1 month.

**Figure 8:**
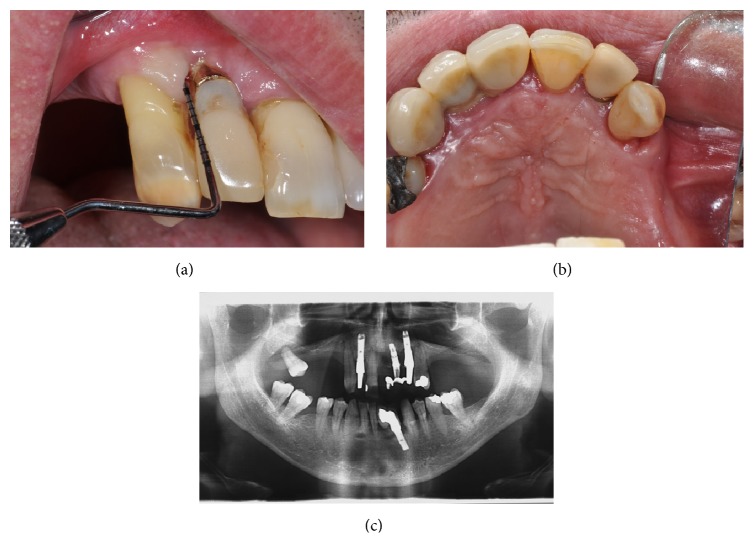
(a, b, c) Clinical and radiological follow-up after 19 months.

At 19-month follow-up, there were no clinical or radiological signs of infections or recurrence (Figures 8(a), 8(b), and 8(c)). A soft tissue defect was evident on the vestibular side. Regenerative surgical therapy was proposed to the patient who, however, refused the treatment.

## 3. Histological Features

**Figure 9:**
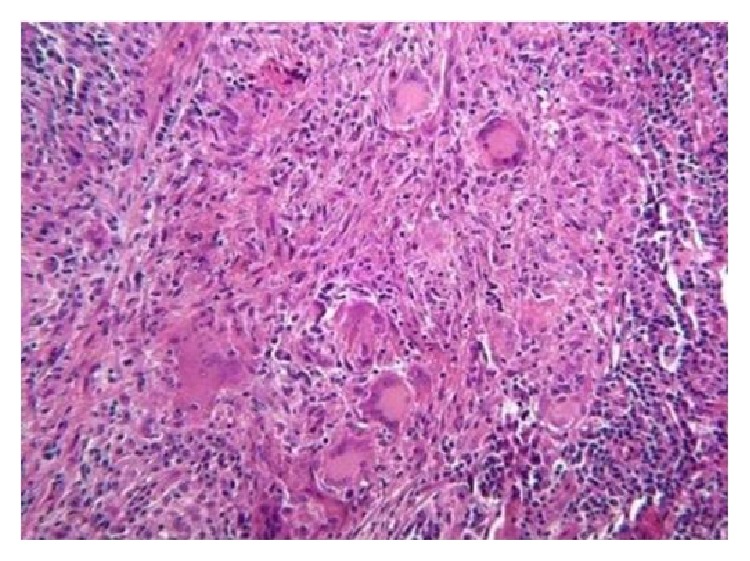
Histologic examination (hematoxylin and eosin ×10).

Histological examination showed the presence of focal clusters of multinucleate giant cells dipped in a richly vascularized connective tissue containing spindle or ovoid mononuclear cells. Giant cells present numerous variations of size, shape, and number of nuclei. A mixed inflammatory infiltrate is also observable in the vascular stroma. The histological section was compatible with the diagnosis of PGCG (Figure 9).

## 4. Discussion

PGCG infrequently arises from peri-implant tissues: only other 15 cases have been described before this one in the English literature [19–29]. The clinical appearance of the lesion associated with dental implants is comparable to that of the lesion associated with natural teeth. Even the preferential localization in the posterior region of the mandible and the higher incidence among females seem to equate to the form arising from peri-implant tissues and the one associated with teeth. In the case herewith reported, the lesion presents less frequent features since it occurred in the anterior area of the upper jaw of a male patient.

The etiopathogenesis of PGCG associated with dental implants is not yet totally clear because the cases reported in the literature are still too few. Since PGCG is a reactive lesion, it is conceivable that some kinds of irritating factors play a determining role in the manifestation of this disease [1, 8–10]. In the reported case, local etiologic factors of the classical form of the PGCG such as plaque, tartar, and gingival inflammation were present since the patient had poor oral hygiene. Systemic etiological factors, such as hyperparathyroidism and addiction to tobacco, were excluded by anamnesis and haematochemical tests. Furthermore, possible etiologic factors of peri-implant diseases in general should be considered. Peri-implantitis is defined as a continuous loss of peri-implant bone tissue associated with a state of inflammation of peri-implant soft tissues that, over time, can lead to the loss of osseous integration and the implant itself [30]. The peri-implantitis features two major types of causal factors: biomechanical factors and infective factors [31]. In the examined case, biomechanical factors are excluded in the aetiology of bone loss around the implant, since the planning and realization of prosthetic therapy were carried out so as not to cause “implant overloading.” On the contrary, the etiological component of infectious type seems to be more likely because of the poor oral hygiene of the patient. Another issue to consider is the possibility that the amount of bone at the implant site was not adequate, and therefore a portion of the fixture had already been exposed since its insertion.

Clinical difficulties associated with this disease result from the necessity of being able to perform the differential diagnosis of PGCG. Other gingival reactive lesions such as pyogenic granuloma and peripheral ossifying fibroma clinically appear very similar to PGCG and thus can be distinguished after histological examination. Pyogenic granuloma's histological appearance is characterized by the presence of a mass of angiomatous tissue with a variable amount of collagen in the connective tissue. Peripheral ossifying fibroma is a reactive lesion arising from the periodontal ligament and microscopically shows a well represented mineralized bony tissue immersed into a stromal fibroblastic proliferation; some authors reported a possible case of PGCG and peripheral ossifying fibroma hybrid lesion, with distinct areas having histological characteristics of both diseases [29].

Central giant cell lesions in advanced stages may erode the bone surface and reach the lining soft tissue; in these cases, radiological evidence of bone resorption is diriment.

In this specific case, having the authors hypothesized the diagnostic phase of the reactive nature of the lesion, they considered that preserving the implant by performing excisional biopsy and accurate curettage of the surrounding bone tissue is possible.

## 5. Conclusion

The correct diagnosis and appropriate surgical treatment of peri-implant giant cell granuloma are very important for proper management of the lesion in order to focus on a correct differential diagnosis with other soft tissues peri-implant diseases and with the aim of preserving the implant prosthetic rehabilitation and preventing recurrences.

## Figures and Tables

**Table 1:** Clinical features of dental implant-associated PGCG cases reported in literature.

Authors	Patient (age)	Location	Area	Dimension	Rx exam	Surgical treatment	Implant loss	Recurrence	Follow-up
Hirshberg et al. [22]	Male (31)	Mandible	Posterior	No data	No data	Excisional biopsy	No	Yes	No data
Hirshberg et al. [22]	Male (69)	Maxilla	Anterior	No data	No data	Excisional biopsy + implant removal	Yes	Yes	No data
Hirshberg et al. [22]	Female (44)	Mandible	Posterior	No data	Bone loss	Excisional biopsy + implant removal	Yes	Yes	No data
Bischof et al. [19]	Female (56)	Mandible	Posterior	No data	Bone loss	Excisional biopsy	No	No	36 months
Scarano et al. [25]	Female (48)	Maxilla	Posterior	1 cm in diameter	Bone loss	Excisional biopsy + soft-tissue graft	No	No data	No data
Cloutier et al. [21]	Male (21)	Mandible	Posterior	2.0 × 1.5 cm	Bone loss	Excisional biopsy + implant removal	Yes	No	12 months
Hernandez et al. [23]	Female (45)	Mandible	Posterior	2 cm in diameter	Bone loss	Excisional biopsy + bone curettage	Yes	Yes	108 months
Hernandez et al. [23]	Female (36)	Maxilla	Posterior	0.8 cm in diameter	Bone loss	Excisional biopsy + implant removal	Yes	Yes	12 months
Hernandez et al. [23]	Female (62)	Mandible	Posterior	2 cm in diameter	Bone loss	Excisional biopsy	No	No	2 months
Ozden et al. [24]	Female (60)	Mandible	Posterior	No data	Bone loss	Excisional biopsy	No	No	12 months
Olmedo et al. [20]	Female (64)	Maxilla	Anterior	0.6 × 0.5 × 0.4 cm	Bone loss	Excisional biopsy + bone curettage	No	No	24 months
Penarrocha-Diago et al. [26]	Female (54)	Mandible	Posterior	2.0 cm in diameter	Bone loss	Excisional biopsy + bone curettage	No	No	12 months
Galindo-Moreno et al. [27]	Male (74)	NA	NA	2.5 × 2.0 cm	No data	Excisional biopsy + bone curettage	No	No	6 months
Brown et al. [28]	Male (46)	Mandible	Posterior	1 cm in diameter	Bone loss	Excisional biopsy + bone curettage	No	Yes	12 months
Ogbureke et al. [29]	Male (44)	Mandible	Posterior	4.5 × 2.5 × 1.5 cm	No data	Excisional biopsy	No	No data	No data
Current report	Male (60)	Maxilla	Anterior	2.0 cm in diameter	Bone loss	Excisional biopsy + bone curettage	No	No	19 months

## References

[1] Katsikeris N., Kakarantza-Angelopoulou E., Angelopoulos A. P. (1988). Peripheral giant cell granuloma: clinicopathologic study of 224 new cases and review of 956 reported cases. *International Journal of Oral and Maxillofacial Surgery*.

[2] Flaitz C. M. (2000). Peripheral giant cell granuloma: a potentially aggressive lesion in children. *Pediatric Dentistry*.

[3] Chaparro Avendaño A. V., Berini Aytés L., Gay Escoda C. (2005). Peripheral giant cell granuloma. A report of five cases and review of the literature. *Medicina Oral, Patologia Oral y Cirugia Bucal*.

[4] Cawson R. A., Binnie W. H., Barrett A. W., Wright J. M. (1999). *Patologia Orale*.

[5] Giansanti J. S., Waldron C. A. (1969). Peripheral giant cell granuloma: review of 720 cases. *Journal of Oral Surgery*.

[6] Bodner L., Peist M., Gatot A., Fliss D. M. (1997). Growth potential of peripheral giant cell granuloma. *Oral Surgery, Oral Medicine, Oral Pathology, Oral Radiology, and Endodontics*.

[7] Nedir R., Lombardi T., Samson J. (1997). Recurrent peripheral giant cell granuloma associated with cervical resorption. *Journal of Periodontology*.

[8] Burkes E. J., White R. P. (1989). A peripheral giant-cell granuloma manifestation of primary hyperparathyroidism: report of case. *The Journal of the American Dental Association*.

[9] Bergdahl L. (1975). Giant cell lesion of the mandible in coincidental hyperparathyroidism and hyperthyroidism. *American Surgeon*.

[10] Wolfson L., Tal H., Covo S. (1989). Peripheral giant cell granuloma during orthodontic treatment. *American Journal of Orthodontics and Dentofacial Orthopedics*.

[11] Sahingur S. E., Cohen R. E., Aguirre A. (2004). Esthetic management of peripheral giant cell granuloma. *Journal of Periodontology*.

[12] Abu Gharbyah A. Z., Assaf M. (2014). Management of a peripheral giant cell granuloma in the esthetic area of upper jaw: a case report. *International Journal of Surgery Case Reports*.

[13] Pammer J., Weninger W., Hulla H., Mazal P., Horvat R. (1998). Expression of regulatory apoptotic proteins in peripheral giant cell granulomas and lesions containing osteoclast-like giant cells. *Journal of Oral Pathology and Medicine*.

[14] Bonetti F., Pelosi G., Martignoni G. (1990). Peripheral giant cell granuloma: evidence for osteoclastic differentiation. *Oral Surgery, Oral Medicine, Oral Pathology*.

[15] Flanagan A. M., Tinkler S. M. B., Horton M. A., Williams D. M., Chambers T. J. (1988). The multinucleate cells in giant cell granulomas of the jaw are osteoclasts. *Cancer*.

[16] Carvalho Y. R., Loyola A. M., Gomez R. S., Araújo V. C. (1995). Peripheral giant cell granuloma. an immunohistochemical and ultrastructural study. *Oral Ddiseases*.

[17] Vered M., Buchner A., Dayan D. (2006). Giant cell granuloma of the jawbones—a proliferative vascular lesion? Immunohistochemical study with vascular endothelial growth factor and basic fibroblast growth factor. *Journal of Oral Pathology and Medicine*.

[18] Hanselaer L., Cosyn J., Browaeys H., De Bruyn H. (2010). Giant cell peripheral granuloma surrounding a dental implant: case report. *Revue Belge de Médecine Dentaire*.

[19] Bischof M., Nedir R., Lombardi T. (2004). Peripheral giant cell granuloma associated with a dental implant. *International Journal of Oral and Maxillofacial Implants*.

[20] Olmedo D. G., Paparella M. L., Brandizzi D., Cabrini R. L. (2010). Reactive lesions of peri-implant mucosa associated with titanium dental implants: a report of 2 cases. *International Journal of Oral and Maxillofacial Surgery*.

[21] Cloutier M., Charles M., Carmichael R. P., Sándor G. K. B. (2007). An analysis of peripheral giant cell granuloma associated with dental implant treatment. *Oral Surgery, Oral Medicine, Oral Pathology, Oral Radiology and Endodontology*.

[22] Hirshberg A., Kozlovsky A., Schwartz-Arad D., Mardinger O., Kaplan I. (2003). Peripheral giant cell granuloma associated with dental implants. *Journal of Periodontology*.

[23] Hernandez G., Lopez-Pintor R. M., Torres J., Vicente J. C. D. (2009). Clinical outcomes of peri-implant peripheral giant cell granuloma: a report of three cases. *Journal of Periodontology*.

[24] Ozden F. O., Ozden B., Kurt M., Gündüz K., Günhan O. (2009). Peripheral giant cell granuloma associated with dental implants: a rare case report. *The International Journal of Oral & Maxillofacial Implants*.

[25] Scarano A., Iezzi G., Artese L., Cimorelli E., Piattelli A. (2008). Peripheral giant cell granuloma associated with a dental implant. a case report. *Minerva Stomatologica*.

[26] Penarrocha-Diago M. A., Cervera-Ballester J., Maestre-Ferrin L., Penarrocha-Oltra D. (2012). Peripheral giant cell granuloma associated with dental implants: clinical case and literature review. *Journal of Oral Implantology*.

[27] Galindo-Moreno P., Hernández-Cortes P., Rios R., Sanchez-Fernández E., camara m., O Valle F. (2013). Immunophenotype of dental implant-associated peripheral giant cell reparative granuloma in a representative case report. *Journal of Oral Implantology*.

[28] Brown A. L., Camargo de Moraes P., Sperandio M., Borges Soares A., Araújo V. C., Passador-Santos F. (2015). Peripheral giant cell granuloma associated with a dental implant: a case report and review of the literature. *Case Reports in Dentistry*.

[29] Ogbureke E. I., Vigneswaran N., Seals M., Frey G., Johnson C. D., Ogbureke K. U. (2015). A peripheral giant cell granuloma with extensive osseous metaplasia or a hybrid peripheral giant cell granuloma-peripheral ossifying fibroma: a case report. *Journal of Medical Case Reports*.

[30] Lindhe J., Berglundh T., Ericsson I., Liljenberg B., Marinello C. P. (1992). Experimental breakdown of peri-implant and periodontal tissues. A study in the beagle dog. *Clinical Oral Implants Research*.

[31] Jovanovic S. A. (1999). Peri-implant tissue response to pathological insults. *Advances in Dental Research*.

